# Twists in the fibrodysplasia ossificans progressiva story challenge and expand our understanding of BMP biology

**DOI:** 10.1172/JCI160773

**Published:** 2022-06-15

**Authors:** Michael T. Collins

**Affiliations:** Skeletal Disorders and Mineral Homeostasis Section, National Institute of Dental and Craniofacial Research, National Institutes of Health, Bethesda, Maryland, USA.

## Abstract

Fibrodysplasia ossificans progressiva (FOP) is an ultrarare, debilitating disease in which heterotopic bone is formed in certain soft tissues. A gain-of-function variant in the cytoplasmic domain of the activin A receptor type I (ACVR1) exists in all patients with FOP. Strikingly, these FOP-causing variants imbue a neofunction to ACVR1 — the ability to recognize activin A as an agonist with bone morphogenic protein–like signaling that leads to heterotopic ossification (HO). These findings are supported by the efficacy of anti–activin A antibodies in preventing HO in FOP mice. This surprising story continues in companion papers in this issue of the *JCI*. Aykul et al. and Lees-Shepard et al. independently found that antibodies against ACVR1, which were being developed as potential therapeutics for FOP, instead caused HO in FOP mice. While this unexpected finding may be the clinical final act for such antibodies, it provides another twist in the unique and evolving FOP story.

## The ACVR1 R206H FOP variant

Fibrodysplasia ossificans progressiva (FOP, identified as OMIM #135100) is an insidious and devastating disease in which there can be massive heterotopic bone formation. Small foci of heterotopic endochondral bone, often stimulated by minor trauma, first appear in childhood. Lesions multiply, coalesce, expand, and mature into a mixture of woven and lamellar bone (1), eventually leading to the patient effectively being locked-in with progressive loss of mobility (2). Early mortality, corresponding to a median survival of 45 years, is most often due to cardiorespiratory failure from heterotopic bone–induced thoracic insufficiency syndrome (3). Treatment for FOP represents a tremendous unmet need; there are no globally approved therapies.

Decades of dedicated care and meticulous research by Fred Kaplan and Eileen Shore eventually identified the cause of FOP as gain-of-function missense variants in the exons encoding the cytoplasmic domain of ACVR1 (also known as activin receptor–like kinase-2, ALK2) (4). The vast majority (95%) of patients with FOP carry the R206H variant. ACVR1 is a type 1 bone morphogenic protein (BMP) receptor that upon BMP binding and hetero-oligomerization with type 2 BMP receptors activates Smad1/5/8 signaling, which in a specific cellular context provides a bone-forming signal. Therefore, it was a logical assumption that the R206H variant, either in a constitutive ligand-independent or a hyper-responsive ligand-dependent fashion, activates an osteogenic program in inducible progenitor cells resident in the tissues that undergo heterotopic ossification (HO) in FOP. Early work supported this hypothesis (5, 6).

## Activin A causes FOP

**Figure 1:**
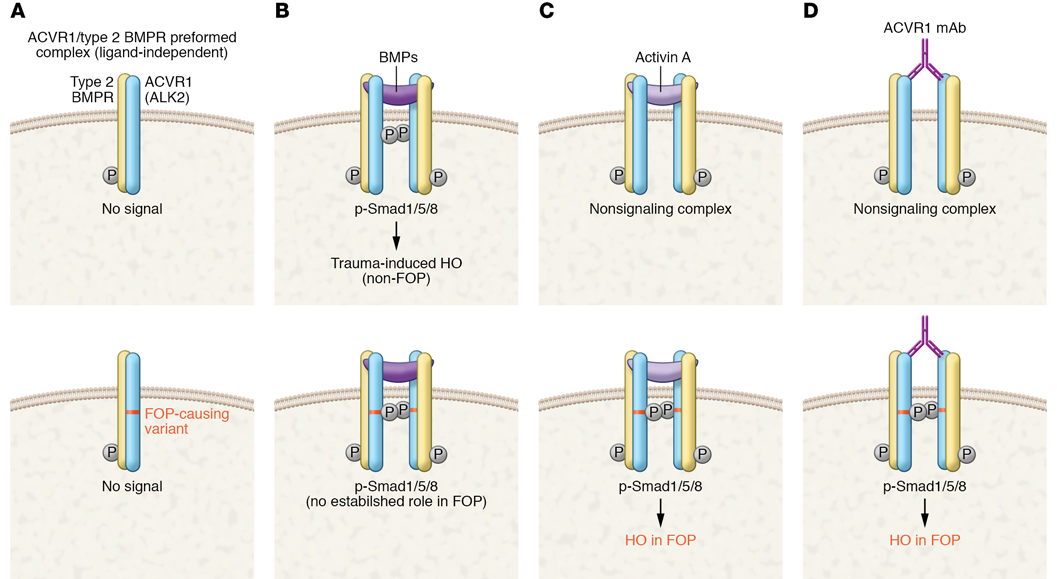
Signaling events and outcomes in WT and R206H ACVR1. (**A**) Heterodimers of ACVR1 (WT or FOP-mutant) and phosphorylated type 2 BMP receptors exist on the membrane in the absence of ligand in preformed heterocomplexes that do not signal (10, 14). (**B**) The binding of BMPs promote heterodimerization of these preformed complexes (WT or FOP-mutant) and result in heterotetramers that lead to the phosphorylation of Smad1/5/8. While there is evidence that WT ACVR1 and type 2 BMP receptor heterotetramers participate in trauma-induced heterotopic ossification (HO), in non-FOP settings (15), there is no evidence that BMP-driven heterotetramers involving FOP-mutant ACVR1 participate in HO in FOP. (**C**) When heterotetramers of WT ACVR1 and corresponding type 2 BMP receptors are formed by activin A binding, a nonsignaling complex results (8). In contrast, a stoichiometrically identical complex formed with FOP-mutant ACVR1 induces phosphorylation of Smad1/5/8 and drives HO in FOP (7). (**D**) Aykul, Huang, et al. (10) found that monoclonal antibodies (mAbs) directed against the extracellular domain of ACVR1, which is the same in WT and FOP ACVR1, led to dimerization and phosphorylation of FOP ACVR1 and the formation of ACVR1/type 2 BMP receptor heterotetramers that were stoichiometrically identical to those formed by ligands (10). With WT ACVR1, the anti-ACVR1/type 2 BMP receptor complexes did not signal, effectively mirroring the nonsignaling complex. However, as independently demonstrated by Aykul, Huang, et al. and Lees-Shepard, Stoessel, et al., the FOP-mutant ACVR1/type 2 BMP heterotetramers resulted in complexes that induced phosphorylation of Smad1/5/8 and drove HO in FOP, much like activin A (10, 11). However, antibody binding alone was insufficient to drive HO in FOP. As in activin A–mediated HO, some, as yet to be identified, factor or signal, probably injury and/or inflammation mediated, is needed to drive HO.

However, this hypothesis appears to be incorrect. With the development of a highly informative mouse model, a team of investigators at Regeneron led by Aris Economides, Vincent Idone, and Sarah Hatsell made the striking discovery that the R206H ACVR1 variant converts ACVR1 to an activin A–responsive, Smad1/5/8-signaling, bone-forming BMP agonist (7). In contrast, under normal physiological conditions, the activin A–bound ACVR1/type 2 receptor complex does not transduce signal; rather, it acts as a nonsignaling complex that opposes BMP signaling by rendering ACVR1 and the type 2 receptors inaccessible for signaling (8). This nonsignaling complex is stoichiometrically identical to complexes formed with activin A–bound R206H ACVR1/type 2 receptor complexes that transduce signal. Hence, FOP-causing mutations convert these nonsignaling complexes to ones that transduce signal, mimicking the complexes formed with BMPs (Figure 1).

This mutation-dependent mimicking ability is striking and, to my knowledge, an unprecedented finding in biology. The R206H variant imparts neofunctional properties to ACVR1, effectively rendering the R206H ACVR1 a neoreceptor. Supporting the veracity of this finding is the efficacy of anti–activin A antibodies in preventing HO in mouse models of FOP (7–9). Further evidence may be derived from clinical studies of an anti-activin antibody in patients with FOP (ClinicalTrials.gov NCT03188666).

## Anti–activin A antibodies cause HO in FOP

The fascinating FOP story takes another twist in this issue of the *JCI*. Given the clear evidence that activin A binding to FOP-variant ACVR1 drives HO in FOP, both Regeneron and David Goldhamer’s laboratory developed anti-ACVR1 antibodies as potential therapeutics for FOP (10, 11). In both cases, in vitro testing in cell lines overexpressing either WT or FOP-mutant ACVR1 confirmed that such antibodies blocked both WT and mutant ACVR1 signaling. But to each group’s surprise, the anti-ACVR1 antibodies promoted the development of HO in FOP mice. Both groups performed a substantial amount of overlapping and complementary work to support and explain this, yet another, surprising phenomenon in FOP.

The Regeneron and Goldhamer groups showed that the FOP-promoting activity of the antibodies was independent of activin A. Goldhamer’s lab, using anti-ACVR1 antibody JAB0505, investigated this phenomenon in a conditional-on mouse model of FOP using two modalities, one in which FOP-mutant ACVR1 was inducibly expressed in all cells, and one where it was expressed only in Tie2-expressing cells that include fibro-adipogenic progenitors (FAPs), a type of muscle-resident progenitor cell that normally participates in muscle repair, but which can also differentiate along the osteogenic lineage to make endochondral bone. In both models, the researchers observed the development of heterotopic bone. They also found that injury-induced HO was substantially delayed, prolonged, and more pronounced in the JAB0505-treated FOP mice, suggesting JAB0505 exacerbates HO, at least in part, by acting to increase the number of R206H-FAPs that ultimately undergo skeletogenic differentiation. However, JAB0505 alone was insufficient to trigger HO in the absence of injury.

Aykul, Huang, et al. (10) developed three anti-ACVR1 monoclonal antibodies (mAb 1, mAb 2, and mAb 3); in vitro, all three demonstrated high affinity for human and mouse ACVR1, lacked binding to related BMP receptors, and blocked Smad1/5/8 signaling in cells overexpressing ACVR1. Like JAB0505, the Regeneron antibodies stimulated R206H ACVR1 signaling and promoted HO in FOP mice. The divergence of mAb 1 in stimulating R206H ACVR1 while inhibiting WT ACVR1 was demonstrated by experiments in WT mice wherein mAb 1 blocked trauma-induced HO, and also inhibited production of hepcidin, the expression of which is largely dependent on BMP6-induced signaling via ACVR1 (12). Through a series of sophisticated and meticulously controlled experiments, Aykul, Huang, et al. (10) discovered an important feature of the anti-ACVR1 antibodies, in that the mechanism of R206H ACVR1 signaling is dependent on receptor homodimerization. Utilizing cells expressing either human WT or R206H ACVR1 in which a small molecule–controlled dimerization domain, DmrB, was fused to their C-termini, they showed that dimerization of ACVR1 led to downstream signaling only with the R206H variant. Further demonstrating relevance of these findings to the clinic, the investigators generated another FOP mouse with a humanized R206H ACVR1 and showed that mAb 1 treatment led to increased HO and reduced iron levels, further evidence that anti-ACVR1 antibodies may not be suitable for the clinic.

## Questions raised

An important finding of the Aykul, Huang, et al. (10) and Lees-Shepard, Stoessel, et al. (11) papers is the implication of an additional factor causing FOP. While three conditions consisting of the R206H ACVR1 variant, activin A or anti-ACVR1 antibody binding, and R206H ACVR1 homodimerization are necessary for HO development in FOP, they are not sufficient. Some other factor introduced by tissue injury, possibly as mild as prolonged everyday wear and tear, is necessary to prime inducible skeletal stem cells (known as FAPs) to respond to osteogenic signals. It is only after FAPs have been brought to this activated state that they can respond to activin A, or to BMPs, as happens in the case of trauma-induced HO. The identity of the signal that induces the activated state in FAPs remains a mystery to be revealed in the next chapter.

Another curious feature of this story is the apparent nonsignaling complex that forms when activin A–bound WT ACVR1 dimerizes with a type 2 BMP. What is the physiological role of this complex? Is it to prevent HO and thus protect the individual from HO? Is it the case that its protective responsibility is overwhelmed in trauma — where BMP levels are so high they outcompete activin A for BMP receptor binding, leading to HO?

ACVR1 does not normally homodimerize. Yet the Regeneron and Goldhamer anti-ACVR1 antibodies appear to cause this interaction, a step effectively converting the so-called “blocking” antibody to an activating ligand in FOP mice. What is it about the structure of R206H ACVR1 that allows for signaling in response to homodimerization by normally nonagonistic ligands? The answer will unfold in the next chapter of the FOP story.

## Conclusion

The changes that convert the ACVR1 in FOP into a bone-forming BMP agonist and the extension of this property to anti-ACVR1 antibodies represents a fascinating example in biology of what the Nobel Laureate Hermann Muller referred to as the creation of a “neomorph” (13), a variant causing a dominant gain of gene function that is different from the normal function. In this case, the receptor neofunction is brought about by activin A binding or anti-ACVR1 antibody–induced homodimerization of the FOP ACVR1. While this neomorph may be the first example of this specific phenomenon in human biology, it is certainly not the only; we just don’t know what the others are yet.

## Version 1 06/15/2022

Electronic publication
